# In Vitro Evaluation of the Neuroprotective Effect of *Panax notoginseng* Saponins by Activating the EGFR/PI3K/AKT Pathway

**DOI:** 10.1155/2020/1403572

**Published:** 2020-07-31

**Authors:** Shuang Wu, Tiantian Yang, Kai Cen, Yihuai Zou, Xiaowei Shi, Dongrui Zhou, Yonghong Gao, Limin Chai, Yizhou Zhao, Yikun Sun, Lingqun Zhu

**Affiliations:** ^1^Key Laboratory of Chinese Internal Medicine of Educational Ministry and Beijing, Dongzhimen Hospital, Beijing University of Chinese Medicine, Beijing, China; ^2^Department of Stomatology, Dongzhimen Hospital, Beijing University of Chinese Medicine, Beijing, China; ^3^Department of Neurology and Stroke Center, Dongzhimen Hospital, Beijing University of Chinese Medicine, Beijing, China; ^4^School of Acupuncture-Moxibustion and Tuina, Beijing University of Chinese Medicine, Beijing, China

## Abstract

**Objective:**

This study investigated whether *Panax notoginseng* saponins (PNS) extracted from *Panax notoginseng* (Bruk.) F. H. Chen played a neuroprotective role by affecting the EGFR/PI3K/AKT pathway in oxygen-glucose deprived (OGD) SH-SY5Y cells.

**Materials and Methods:**

Different groups of OGD SH-SY5Y cells were treated with varying doses of PNS, PNS + AG1478 (a specific inhibitor of EGFR), or AG1478 for 16 hours. CCK8, Annexin V-FITC/PI apoptosis analysis, and LDH release analysis were used to determine cell viability, apoptosis rate, and amounts of LDH. Quantitative real-time PCR (q-RT-PCR) and western blotting were used to measure mRNA and proteins levels of p-EGFR/EGFR, p-PI3K/PI3K, and p-AKT/AKT in SH-SY5Y cells subjected to OGD.

**Results:**

PNS significantly enhanced cell viability, reduced apoptosis, and weakened cytotoxicity by inhibiting the release of LDH. The mRNA expression profiles of EGFR, PI3K, and AKT showed no difference between model and other groups. Additionally, ratios of p-EGFR, p-PI3K, and p-AKT to EGFR, PI3K, and AKT proteins expression, respectively, all increased significantly.

**Conclusions:**

These findings indicate that PNS enhanced neuroprotective effects by activating the EGFR/PI3K/AKT pathway and elevating phosphorylation levels in OGD SH-SY5Y cells.

## 1. Introduction

Stroke is the second leading cause of death worldwide [1] but is the leading cause of death in China [2]. The incidence of ischemic stroke is more than 75% in stroke patients [3]. The optimal treatment time after acute stroke is limited, resulting in a high disability rate [4]. Hence, much attention has been paid to the regeneration and remodeling of neurons to restore nerve function.

Over many years, the efficacy of traditional Chinese medicine (TCM) has slowly been accepted by many countries. The active ingredients extracted from Chinese herbs are also being extensively studied. *Panax notoginseng* saponins (PNS) are extracted from the root or rhizome of the herb *Panax notoginseng* (Bruk.) F. H. Chen (*Araliaceae*). PNS has been widely used to treat cerebrovascular and cardiovascular diseases, including ischemic stroke and coronary artery disease [5, 6]. However, the mechanism of PNS in the treatment of ischemic stroke is poorly understood. We have previously shown that PNS reduced the expression of Nogo-A, NgR, p75, and RhoA, which promoted the recovery of nerve function in rats and SH-SY5Y cells [7, 8]. In addition to inhibitory pathways, there are also factors that promote the recovery of nerve function after ischemic stroke. Epidermal growth factor receptor (EGFR) activation acts on several downstream signaling pathways, promoting angiogenesis, cell proliferation, differentiation, migration, and antiapoptosis [9]. One of the downstream signaling pathways of EGFR is the phosphatidylinositol 3-kinase (PI3K)/protein kinase B (AKT) pathway [10]. Activation of tissue plasminogen activator (tPA) associated EGFR-linked signaling can reduce brain injuries including stroke [11]. A phosphorylation cascade after EGFR dimerization leads to the activation of downstream PI3K/AKT signaling, which plays a vital role in the occurrence and development of various tumors [12–14]. EGFR was transactivated after ischemic stroke and promoted regeneration of neuronal morphology and function mainly by activating the PI3K/AKT pathway [10]. However, the mechanism by which the EGFR/PI3K/AKT pathway acts against neuronal injury remains to be elucidated.

## 2. Materials and Methods

### 2.1. Cells and Cell Culture

A human female neuroblastoma cell line (SH-SY5Y) [15] was purchased from the National Infrastructure of Cell Line Resource (Beijing, China). Cells were grown in Roswell Park Memorial Institute 1640 Medium (1×) (RPMI 1640, 11875093; Gibco, USA) supplemented with 10% fetal bovine serum (FBS, 10099141; Gibco, USA) and 1% penicillin-streptomycin (15140122; Gibco, USA) in an automatic CO_2_ incubator (37°C, 5% CO_2_; MCO20AIC; Sanyo, Japan). Cells are passaged or plated at 80% confluency.

### 2.2. Establishment of an Oxygen-Glucose Deprivation (OGD) Cell Model

SH-SY5Y cells were cultured in an automatic CO_2_ incubator under normal conditions. After 24 hours, the cell culture medium was replaced with glucose-free RPMI 1640 (11879020; Gibco, USA) without serum. Cells were then incubated in a trigas incubator (37°C, 5% CO_2_, 1% O_2_, 94% N_2_, MiniGalaxy A500; RSBiotech, UK) for 6 different periods of time (4, 8, 12, 16, 20, 24 hours). The most suitable culture time was selected according to the cell viability determined by using CCK8.

### 2.3. Drug and Optimum Drug Concentration

**Figure 1:**
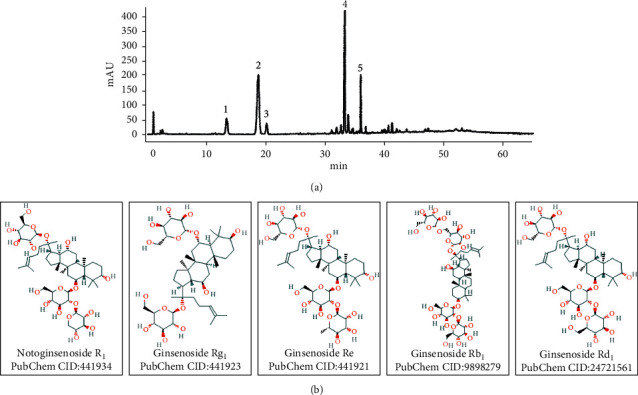
The chemical fingerprint and five major formula structures of PNS. (a) The chemical fingerprint of PNS was extracted from the Chinese pharmacopoeia 2015 edition. (b) Five major formula structures of PNS excerpted from the NCBI website.

The major active components of PNS (Batch No. 16BB205; KPC Pharmaceuticals, Inc, China) include 9.8% (v/v) Notoginsenoside R_1_, 38.4% (v/v) Ginsenoside Rg_1_, 5.4% (v/v) Ginsenoside Re, 34.8% (v/v) Ginsenoside Rb_1_, and 4.7% (v/v) Ginsenoside Rd, with the total pharmaceutical concentration of 93.0% (v/v) in the PNS fraction. The chemical fingerprint of PNS and the formula structures of five major components are shown in Figure 1. Methods to determine the optimal dosage of PNS are based on our previous study [8]. To determine the optimal concentration of PNS, the cell suspension (100 *μ*l/well) with 1 × 10^4^ cells was added to 96-well plates (Corning, USA). PNS were dissolved in a glucose-free RPMI 1640 medium. Seven different PNS concentrations (40, 80, 160, 320, 640, 1280, 2560 *μ*g/mL) were added to OGD injury SH-SY5Y cells. The control groups were not treated with drug intervention. The optimum concentration of PNS was determined according to the cell viability determined by using CCK8. The optimal concentration of AG1478 (S2728; Selleck, USA) was determined as 1 *μ*M by referring to a published study [16].

### 2.4. Experimental Groups

The cell groups were divided into normal, OGD model, PNS, AG1478, and PNS plus AG1478 (P + A) groups according to the drugs administered.

### 2.5. CCK8 Cell Viability Assay

SH-SY5Y cell viability assays were performed using CCK8 assay kits (CK04-500; Dojindo, Japan) according to the manufacturer's instructions. In brief, after different treatments, 10 *μ*L of CCK8 solution was added to cells in a 96-well plate incubated in an automatic CO_2_ incubator (37°C, 5% CO_2_) for 2 hours. Then, the optical density (OD) of cells was measured at 450 nm in a microplate reader (MULTISKAN MK3; Thermo, USA). All experiments were repeated over three times.

### 2.6. Annexin V-FITC/PI Assay for Apoptosis

SH-SY5Y cell apoptosis was detected using an Annexin V-FITC/PI apoptosis detection kit (KGA107; KeyGEN BioTECH, China). SH-SY5Y cells were seeded in 6-well plates and exposed to different media in a trigas incubator (37°C, 5% CO_2_, 1% O_2_, 94% N_2_, MiniGalaxy A500; RSBiotech, UK) for 16 h. Then, the cells were detached with 0.25% EDTA-free trypsin (15050065; Gibco, USA) and centrifuged in a cryogenic centrifuge (Thermo Scientific Heraeus Pico and Fresco 17; Thermo, USA) with a centrifugal force of 181.1 ×*g*. Cells were then resuspended in 500 *μ*L binding buffer. Five microliters of Annexin V-FITC and PI were then added to the cell suspension and incubated in the dark at room temperature for 15 minutes. The experiments were done in triplicate and repeated more than three times.

### 2.7. Lactate Dehydrogenase (LDH) Release Assay for Cytotoxicity

The cytotoxicity of SH-SY5Y cells was detected using a lactate dehydrogenase assay kit (A020-2; Nanjing Jiancheng Bioengineering Institute, China) according to the manufacturer's instructions. Absorbance (OD) was measured with a microplate reader (Varioskan Flash; Thermo, USA) at 450 nm. The activity of LDH was calculated from the OD values. All procedures were repeated at least three times.

### 2.8. Quantitative Real-Time PCR (qRT-PCR)

Total RNA was extracted from SH-SY5Y cells cultured in 6-well plates (Corning, USA) using TRI Reagent (T9424; Sigma, USA). Agarose gel electrophoresis and spectrophotometry (Nanogrop2000; Thermo, USA) were used to determine the quantity and quality of total RNA. First-strand cDNA was synthesized using a reverse transcription kit (A5001; Promega, USA) and amplified by PCR with a real-time PCR kit (A6001; Promega, USA). All procedures were repeated at least three times.

The primer sequences used for qRT-PCR were as follows: GAPDH, FWD 5′-CCTCCTGAACTTGAGGCAGTTT-3′, Rev 5′-TGTATTGTAACCAGTCATCA-GCA-3′, EGFR FWD 5′-TGGGTGGCCTCCTCTTCATA-3′, Rev 5′-GGTGTG-AGAGGTTCCACGAG-3′, PI3K FWD 5′-ATCGACCTACACTTGGGGGA-3′, Rev 5′-CAATATCTTCTGGCCGGGCT-3′, AKT FWD 5′-TGGAGTGTGTGGA-CAGTGAAC-3′, Rev 5′-AGGTACAGATGATCCATGCGG-3′. Reactions were performed on the iCycler Thermal Cycler 96-Well Thermal Sealing Ring (iCycler iQ; Bio-Rad, USA). Expression levels of mRNAs were calculated using the 2^−△△CT^ method.

### 2.9. Western Blotting

Proteins were isolated from cultured SH-SY5Y cells. In brief, SH-SY5Y cells were rinsed twice with phosphate buffer saline (1×) (PBS, SH30256.01B, Hyclone), detached with EDTA-free 0.25% trypsin, and centrifuged. Total protein concentrations were determined with a BCA protein assay kit (P0012S; Beyotime, China). Western blot analysis of total protein extracted from SH-SY5Y cells was described previously. The prestained dual color protein molecular weight marker (P0068) was purchased from Beyotime. The antibodies included p-PI3K (ab182651, 1 : 2000), PI3K (ab74136, 1 : 1000), p-AKT (ab38449, 1 : 2000), AKT (ab8805, 1 : 2000), EGFR (ab52894, 1 : 2000), GAPDH (ab9485, 1 : 2500) from Abcam and Lingo-1 (#48389, 1 : 1000), and p-EGFR (#4407, 1 : 1000) from CST. Gel Pro software was used to analyze images. All experiments were performed at least three times.

### 2.10. Statistical Analysis

Statistical analysis of the data was conducted using SPSS 17.0 software. All data are expressed as mean ± standard error (SEM) and were analyzed by one-way ANOVA, the LSD post hoc test when equal variance was assumed or by the Kruskal–Wallis test when equal variance was not assumed. *P* < 0.05 indicated a statistically significant difference.

## 3. Results

### 3.1. Optimal Oxygen Deprivation Culture Time for SH-SY5Y Cells and the Optimum Concentration of PNS

**Figure 2:**
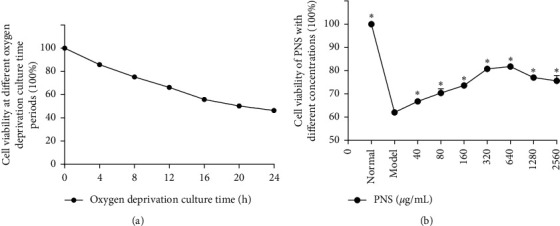
The optimal oxygen deprivation culture time and the optimum concentration of PNS on cell viability in SH-SY5Y cells. (a) The optimal oxygen deprivation culture time of SH-SY5Y cells was 16 h, as determined by cell viability (*n* = 8). (b) The optimal concentration of PNS was 640 *μ*g/mL according to the results of CCK8. Values are expressed as the mean ± SEM (*n* = 8), ^*∗*^*P* < 0.01 vs. model group.

The cell viability measured by CCK8 decreased gradually with the increase of hypoxia time. After 16 h of oxygen deprivation culture, the cell survival rate was 55.9%, which met the requirements for continuing the experiment under these conditions (Figure 2(a)). The PNS concentration was 40 *μ*g/mL, and the cell viability was 66.8%, which was significantly higher than that of the model group (66.8 ± 0.6 vs. 62.0 ± 1.0, *P* < 0.01). With the increase of PNS concentration, the cell viability of OGD SH-SY5Y cells also increased gradually. When the PNS concentration was 640 *μ*g/mL, the cell viability was as high as 81.8% (81.8 ± 1.1 vs. 62.0 ± 1.0, *P* < 0.01). However, as PNS concentration continued to increase, the cell viability began to decline compared to the model group (77.0 ± 0.6 vs. 62.0 ± 1.0, *P* < 0.01; 75.6 ± 2.3 vs. 62.0 ± 1.0, *P* < 0.01). In summary, the optimal concentration of PNS was 640 *μ*g/mL. (Figure 2(b)).

### 3.2. Cell Viability of OGD-Injured SH-SY5Y Cells after Treatment

**Figure 3:**
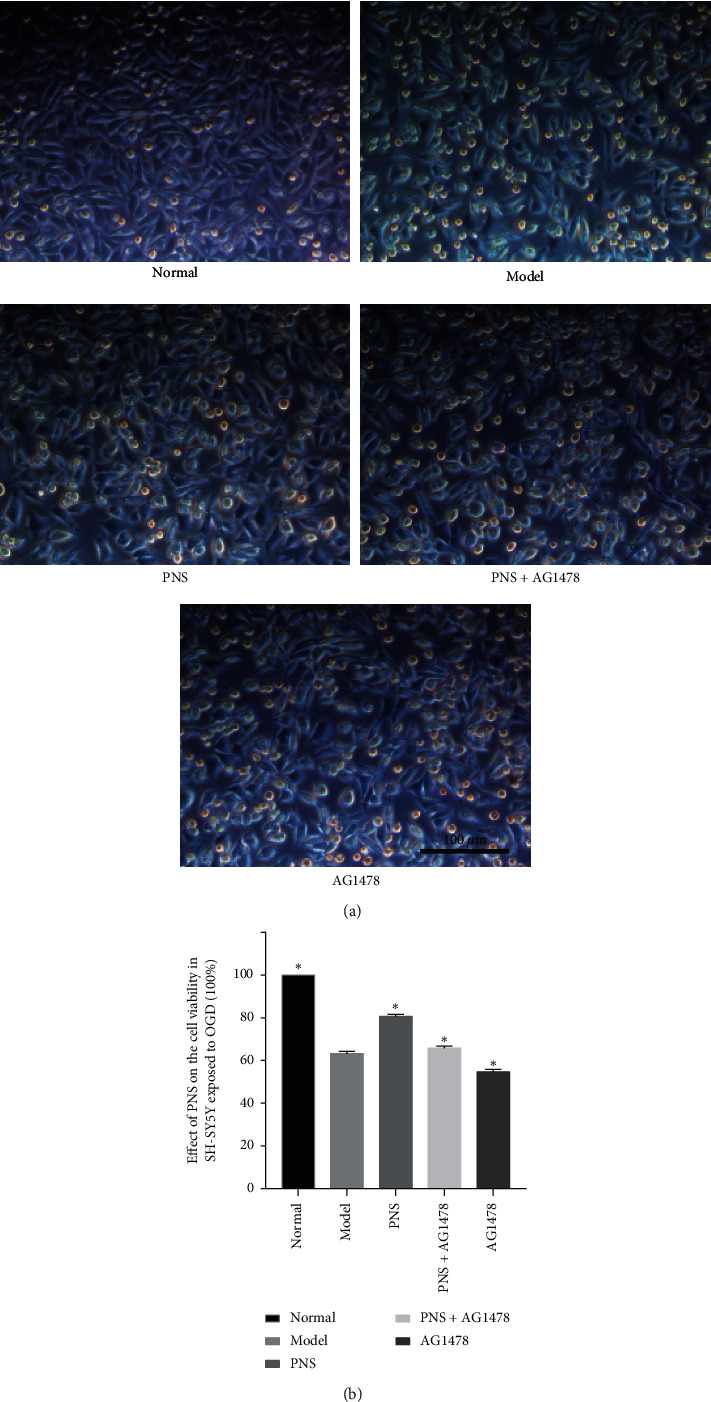
Morphology and cell viability of SH-SY5Y cells after treatment. (a) The morphology of SH-SY5Y cells in each group was observed by inverted microscopy (magnification, 200X, scale bar, 100 *μ*m). The normal group cells adhered to the well and grew in a fusiform or polygonal shape, with round cell bodies and fine synapses. The cell bodies and synapses of the model group were atrophic, and a large number of floating dead cells appeared in the culture dish. There were fewer dead cells in the PNS group and P + A group than in the model group, especially the PNS group. (b) Cell viability was significantly improved in the PNS group compared with the model group (*P* < 0.01) and significantly reduced in the AG1478 group compared with the model group (*P* < 0.01). The cell viability of the P + A group was significantly lower than that of the PNS group (*P* < 0.01). Values are expressed as the mean ± SEM (*n* = 8). ^*∗*^*P* < 0.01 vs. model group. ^#^*P* < 0.01 vs. PNS group. 8017563

The protective effect of PNS on OGD-injured SH-SY5Y cells was detected by the CCK8 assay. OGD caused severe damage to SH-SY5Y cells and the survival rate decreased to about 63.3%. Cell viability was significantly reduced in the model group compared with the normal group (63.3% ± 1.0% vs. 100% ± 0.0%, *P* < 0.01). After treatment with PNS, cell viability was significantly elevated compared with the model group (80.6% ± 1.0% vs. 63.3% ± 1.0%, *P* < 0.01). When AG1478 was used alone, the cell survival rate was significantly lower than that of the model group (54.6% ± 1.2% vs. 63.3% ± 1.0%, *P* < 0.01). The effect of PNS was weakened when used in combination with AG1478 (65.9% ± 0.9% vs. 80.6% ± 1.0%, *P* < 0.01) (Figure 3).

### 3.3. Annexin V-FITC/PI Assay for Apoptosis

**Figure 4:**
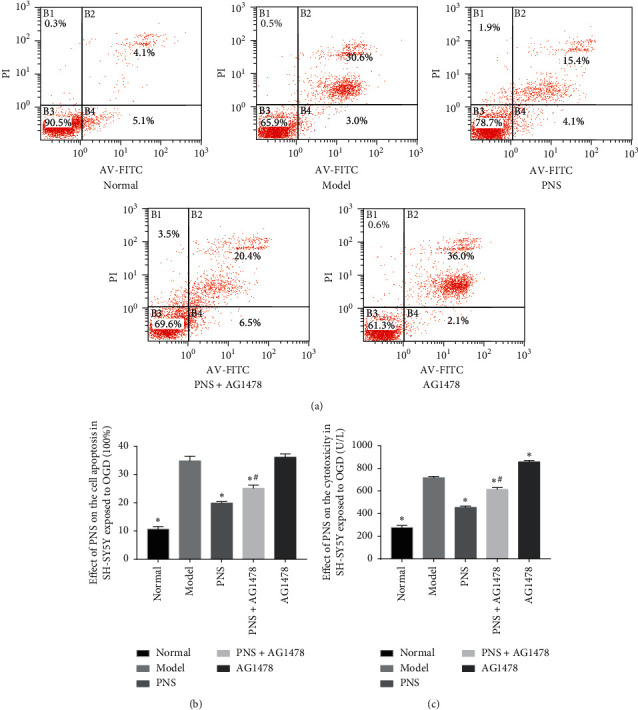
Apoptosis and cytotoxicity of SH-SY5Y cells after treatment. The apoptosis rate is the sum of B2 and B4. (a) Following treatment with PNS and P + A, apoptosis rates were significantly reduced (*P* < 0.01). The apoptosis rate of the P + A group was significantly higher than that of the PNS group (*P* < 0.05). Values are expressed as the mean ± SEM (*n* = 3). ^*∗*^*P* < 0.01 vs. model group. ^#^*P* < 0.05 vs. PNS group. (b) The bar graph represents the apoptosis rate of each group. (c) The bar graph shows the cytotoxicity of the five groups. In brief, PNS and P + A treatment reduced cytotoxicity of OGD SH-SY5Y cells compared with the model group (*P* < 0.01). The cytotoxicity of the P + A group was significantly enhanced compared with the PNS group (*P* < 0.01). Values are expressed as the mean ± SEM (*n* = 6). ^*∗*^*P* < 0.01 vs. model group. ^#^*P* < 0.01 vs. PNS group.

Apoptosis of SH-SY5Y cells after OGD and drug intervention was tested using the Annexin V-FITC/PI assay. OGD significantly increased the apoptosis rate of SH-SY5Y cells in the model group compared with the normal group (34.83 ± 1.65 vs. 10.63 ± 0.94, *P* < 0.01). The apoptosis rate of the PNS group was significantly lower than that in the model group (19.90 ± 0.50 vs. 34.83 ± 1.65, *P* < 0.01). However, when PNS and AG1478 were combined, cell apoptosis was significantly increased compared with that in the PNS group (25.10 ± 1.14 vs. 19.90 ± 0.50, *P* < 0.05) (Figures 4(a) and 4(b)).

### 3.4. Lactate Dehydrogenase (LDH) Release Assay for Cytotoxicity

Cytotoxicity of OGD SH-SY5Y cells was detected by an LDH release assay. OGD severely damaged SH-SY5Y cells and significantly enhanced their cytotoxicity in comparison with the normal group (717.86 ± 11.02 vs. 277.45 ± 18.21, *P* < 0.01). PNS treatment resulted in significantly reduced cytotoxicity of OGD SH-SY5Y cells compared with the model group (454.66 ± 12.08 vs. 717.86 ± 11.02, *P* < 0.01). P + A treatment resulted in increased cytotoxicity of SH-SY5Y cells compared with the PNS group (614.29 ± 15.91 vs. 454.66 ± 12.08, *P* < 0.01) (Figure 4(c)).

### 3.5. EGFR, PI3K, and AKT mRNA Levels in OGD-Injured SH-SY5Y Cells after Treatment

**Figure 5:**
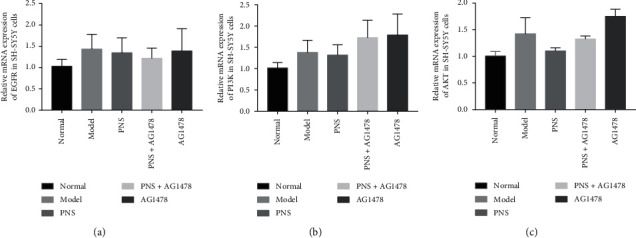
mRNA levels of (a) EGFR, (b) PI3K, and (c) AKT in SH-SY5Y cells after treatment. Bar graphs represent mRNA levels of EGFR, PI3K, and AKT. There were no significant differences in EGFR, PI3K, AKT mRNA levels among groups. Values are expressed as the mean ± SEM (*n* = 3).

There were no significant differences in EGFR, PI3K, AKT mRNA profiles between the model group and the other groups (*P* > 0.05) (Figure 5).

### 3.6. Ratios of Phospho-EGFR/EGFR, Phospho-PI3K/PI3K, and Phospho-AKT/AKT Protein Levels in SH-SY5Y Cells following Treatment

**Figure 6:**
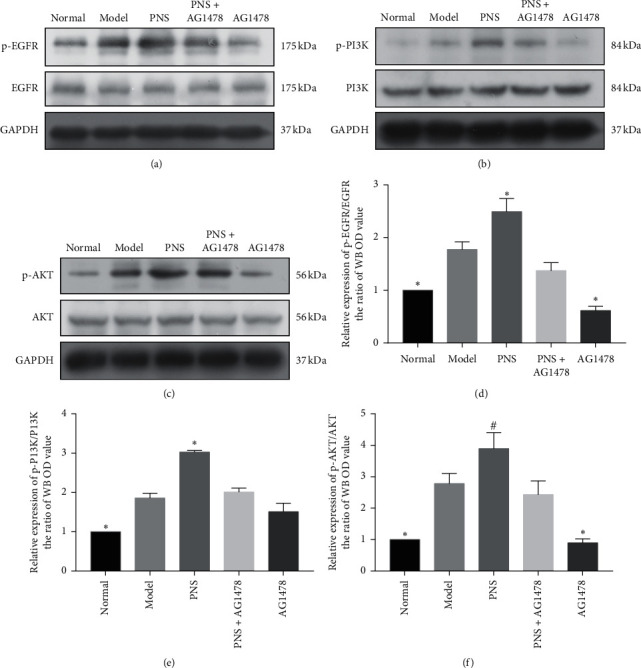
Phospho-EGFR, phospho-PI3K, and phospho-AKT protein levels in SH-SY5Y cells following treatment.

Phospho-EGFR/EGFR, phospho-PI3K/PI3K and phospho-AKT/AKT protein levels in SH-SY5Y cells were determined by western blotting. Ratios of phospho-EGFR/EGFR, phospho-PI3K/PI3K, and phospho-AKT/AKT were significantly upregulated in the model group compared with the normal group (1.77 ± 0.15, 1.86 ± 0.12, 2.78 ± 0.32 vs. 1.00 ± 0, *P* < 0.01). Treatment with PNS significantly increased phospho-EGFR/EGFR, phospho-PI3K/PI3K, and phospho-AKT/AKT compared with the model group (2.49 ± 0.25 vs. 1.77 ± 0.14, *P* < 0.01; 3.03 ± 0.04 vs. 1.86 ± 0.12, *P* < 0.01; 3.89 ± 0.51 vs. 2.78 ± 0.32, *P* < 0.05) (Figure 6).

Protein levels of phospho-EGFR/EGFR, phospho-PI3K/PI3K, and phospho-AKT/AKT in SH-SY5Y cells were detected by western blotting (Figures 6(a)–6(c)). Bar graphs represent the statistical analysis of phospho-EGFR/EGFR, phospho-PI3K/PI3K, and phospho-AKT/AKT (Figures 6(d)–6(f)). In brief, the phosphorylation levels of EGFR, PI3K, and AKT in the PNS group were significantly increased compared with the model group (*P* < 0.01 or *P* < 0.05). Values are expressed as the mean ± SEM (*n* = 3). ^*∗*^*P* < 0.01 vs. model group. ^#^*P* < 0.05 vs. model group.

## 4. Discussion

The main cause of death and disability after ischemic stroke is caused by decreased neuroprotection and neuroregeneration in the ischemic area [17]. Free radicals and calcium overload are widely studied mechanisms in the pathological processes of ischemic damage, especially at the stage of ischemia reperfusion [18–20]. The brain generates more free radicals than any other organs, resulting in free radical damage of brain tissue [21]. On the other hand, under the condition of insufficient glucose and oxygen, the normal ionic gradients of neurons are destroyed, leading to cellular calcium overload [22]. The excessive intracellular Ca^++^ leads to the activation of excitotoxicity, triggering cell death [23]. In addition, the inhibitory environment for nerve growth leads to the limited of neuroregeneration [24, 25]. Identification of effective targets to promote the regeneration and remodeling of damaged nerves is a focus of research on cerebral infarction. In this study, we found that PNS played a role in promoting nerve regeneration and protection by activating EGFR/PI3K/AKT signaling.

Epidermal growth factor (EGF) was first identified in newborn mice in 1962 [26]. Twenty years later, the epidermal growth factor receptor (EGFR) was isolated and purified [27]. EGFR, also called ErB1 and HER1, is a member of the ErbB family which has tyrosine kinase activity [28]. As a transmembrane tyrosine kinase receptor, EGFR exists in immature astrocytes and disappears as the astrocytes mature [29, 30]. The EGFR structure comprises three parts: the extracellular glycosylated ligand recognition domain, a hydrophobic transmembrane segment, and the intracellular portion containing a highly conserved tyrosine kinase sequence [28]. After transactivation, EGFR stimulates downstream signaling cascades to protect cells or to promote cell growth [10, 31, 32]. PI3K/AKT is one of the most representative signaling pathways activated after phosphorylation of EGFR [10, 33]. PI3K is a phospholipid kinase involved in cell growth, proliferation and differentiation [34]. Kinase protein kinase B (PKB, also called AKT) is a serine-threonine protein. It is activated by PI3K to play roles in cell growth, cell-cycle regulation, and inhibition of apoptosis [35, 36].

The EGFR/PI3K/AKT pathway is extensively studied in the field of cancer biology. However, it is also of vital importance in nervous system diseases, such as ischemic stroke [34]. The traditional Chinese medicine, PNS, has been widely used in the treatment of cerebrovascular disease because of its known composition and potent effects [37]. PNS has been investigated from different perspectives. We previously showed that PNS exerted neuroprotective and anti-inflammatory roles by inhibiting NgR1, RhoA, ROCK2, IL-1*β,* and TNF-*α* [8, 38]. In order to further investigate the mechanism by which PNS promotes nerve regeneration or reduces neural apoptosis, the EGFR/PI3K/AKT signaling pathway was studied. In this study, we found that PNS protected OGD SH-SY5Y cells by activating the EGFR/PI3K/AKT signaling pathway.

SH-SY5Y cells are human female neuroblastoma cells that are commonly used to mimic the mechanism of cerebral infarction in vitro [39]. Cell viability is an important indicator to evaluate cell growth, proliferation, and differentiation. CCK8 is the most commonly used method to detect cell viability [40]. Our results show that the cell viability in the model group was significantly reduced compared with the normal group. After treatment with PNS, cell viability was significantly elevated compared with the model group, and the effect of PNS was weakened when combined with AG1478. AG1478 is a specific inhibitor of EGFR [41, 42] and abolished the neuroprotective effects of PNS. Obviously, the cell survival rate when AG1478 was used alone was significantly lower than that of the model group. We also observed the morphology of SH-SY5Y cells by inverted microscopy and found fewer floating dead cells in the PNS group than in the model group. These results indicated that the OGD modeling of SH-SY5Y cells was successful and that PNS played a protective role in OGD SH-SY5Y cells.

Apoptosis, also called programmed cell death [43], normally occurs in multicellular organisms [44]. Annexin V-FITC/PI assay was used to monitor the apoptotic rate of each group. LDH is a glycolytic enzyme that exists in the cytoplasm of normal cells. When cells are damaged, cell membrane permeability increases and LDH exudes to the outside of cells. Therefore, the degree of cell damage can be detected and determined in the cell supernatant. The apoptosis rate and LDH activity in the cell culture medium of SH-SY5Y cells after OGD injury were significantly increased compared with the normal group. PNS significantly reduced the apoptosis rate and LDH activity of OGD SH-SY5Y cells compared with the model group. AG1478 weakened the protective effect of PNS on OGD SH-SY5Y cells. These results show that PNS can protect OGD SH-SY5Y cells by reducing apoptosis and cell membrane damage.

Quantitative real-time PCR, with its advantages of sensitivity, specificity, and reproducibility, has been widely used to detect mRNA levels [45]. There were no differences in mRNA levels of EGFR, PI3K, and AKT among the five groups. This indicated that PNS and AG1478 had no effect on EGFR, PI3K, or AKT mRNA levels in OGD SH-SY5Y cells. One of the most popular used laboratory techniques in researches is western blotting [46, 47]. The ratios of p-EGFR to EGFR, p-PI3K to PI3K, and p-AKT to AKT were significantly higher in the model group than in the normal group. Compared with the model group, the ratios of p-EGFR to EGFR, p-PI3K to PI3K, and p-AKT to AKT in the PNS group were significantly increased. The ratio of p-EGFR to EGFR in the AG1478 group was significantly reduced compared with that in the model group. These results indicate that the phosphorylation levels of EGFR, PI3K, and AKT were increased in OGD-injured SH-SY5Y cells. Interestingly, the phosphorylation levels of EGFR, PI3K, and AKT were higher after PNS treatment. Meanwhile, AG1478 significantly inhibited the phosphorylation and the activation of EGFR, which is consistent with results from other studies [48, 49].

## 5. Conclusions

PNS improved cell viability and played a neuroprotective role by inhibiting apoptosis and cell membrane damage in SH-SY5Y cells exposed to OGD. Furthermore, PNS exerted neuroprotective effects by activating the EGFR/PI3K/AKT pathway and elevating EGFR/PI3K/AKT phosphorylation levels in OGD SH-SY5Y cells.

## Data Availability

All data generated or analyzed during this study are included in this published article (and its supplementary information files).

## Additional Points

*Highlights.*The activation of EGFR/PI3K/AKT pathway maintained the activity and inhibited the apoptosis of oxygen-glucose deprived SH-SY5Y cells in vitro. The EGFR/PI3K/AKT pathway was activated of oxygen-glucose deprived SH-SY5Y cells after *Panax notoginseng* saponins treatment. The increase of phosphorylation levels of EGFR, PI3K, and AKT is involved in the neuroprotective effect of *Panax notoginseng* saponins on oxygen-glucose deprived SH-SY5Y cells.

## Supplementary Materials

Supplementary Materials The supplementary material files include all the experimental data used in the manuscript, such as the optimal oxygen deprivation culture time, the optimum concentration of PNS, morphology, cell viability, apoptosis and LDH release of SH-SY5Y cells (Documents 2–8). Meanwhile, results and figures of q-RT PCR and western Blot are also included (Documents 9–11). In addition, document 1 is the test report of PNS.
