# Supplementary material for: In Vitro Evaluation of the Neuroprotective Effect of Panax notoginseng Saponins by Activating the EGFR/PI3K/AKT Pathway
Source: Evid Based Complement Alternat Med. 2020 Jul 31;2020:1403572. doi: 10.1155/2020/1403572 (PMC7415117; doi:10.1155/2020/1403572)
Supplement: Supplementary Materials — The supplementary material files include all the experimental data used in the manuscript, such as the optimal oxygen deprivation culture time, the optimum concentration of PNS, morphology, cell viability, apoptosis and LDH release of SH-SY5Y cells (Documents 2–8). Meanwhile, results and figures of q-RT PCR and western Blot are also included (Documents 9–11). In addition, document 1 is the test report of PNS. [file 1403572.f1.pdf]

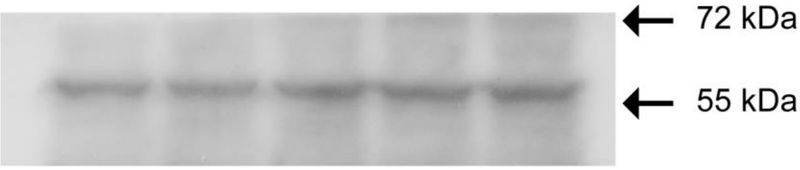

AKT-1

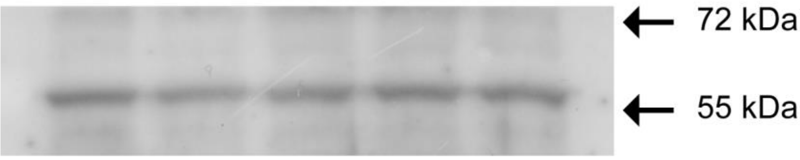

AKT-2

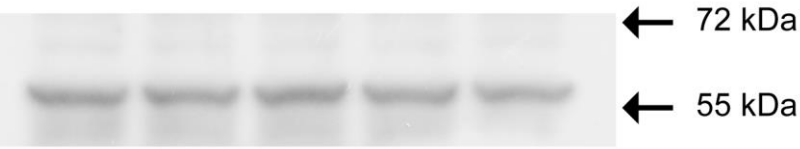

AKT-3

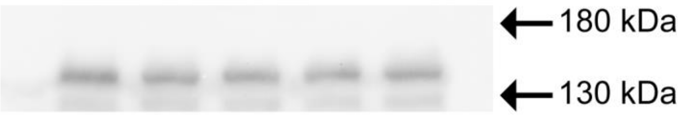

EGFR-1

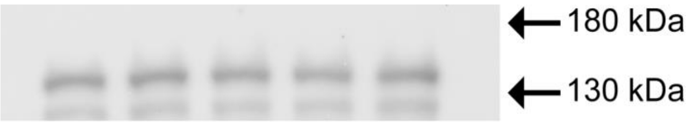

EGFR-2

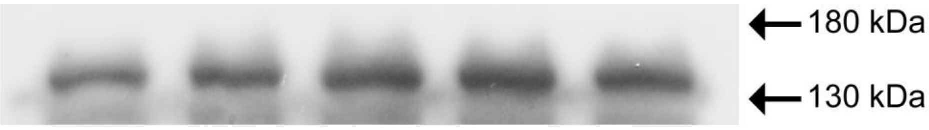

EGFR-3

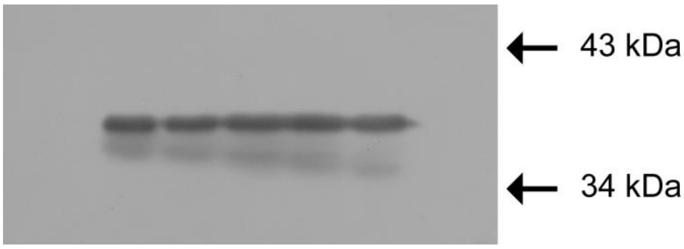

GAPDH-1

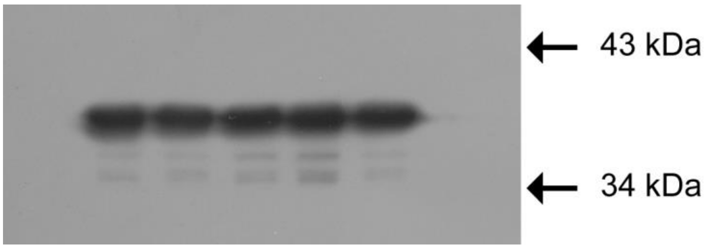

GAPDH-2

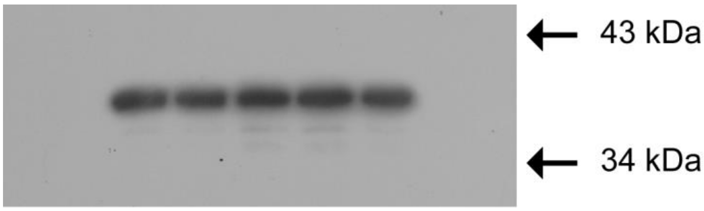

GAPDH-3

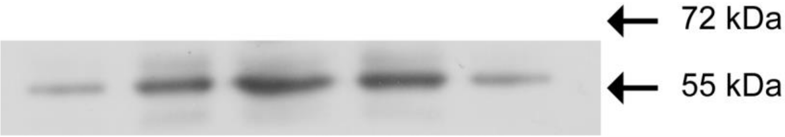

p-AKT-1

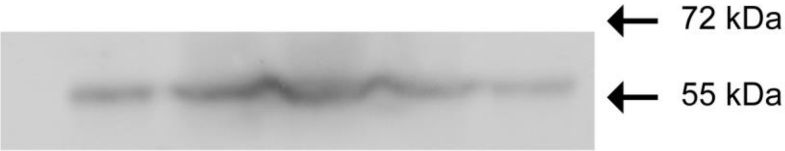

p-AKT-2

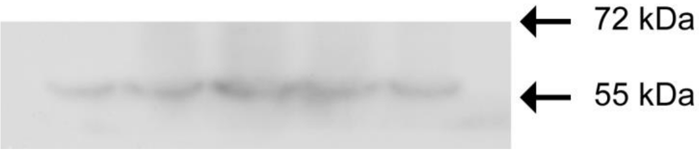

p-AKT-3

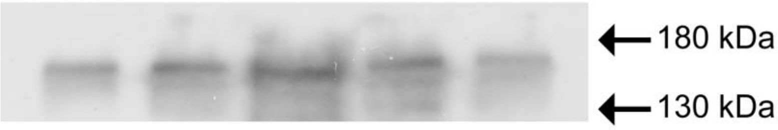

p-EGFR-1

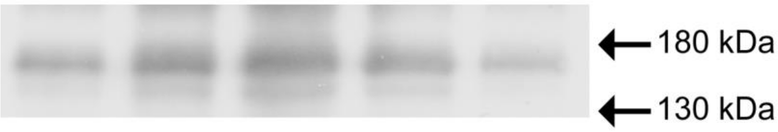

p-EGFR-2

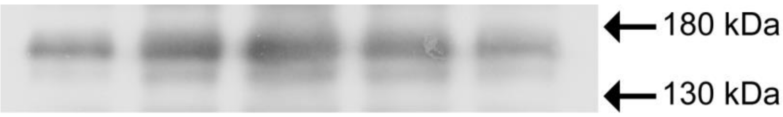

p-EGFR-3

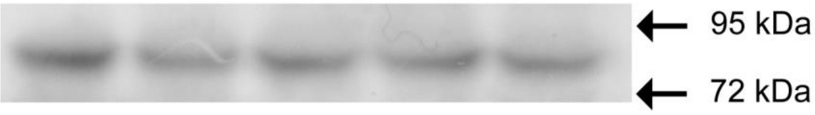

PI3K-1

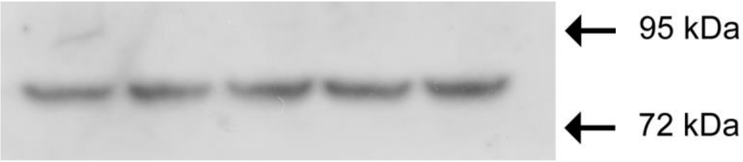

PI3K-2

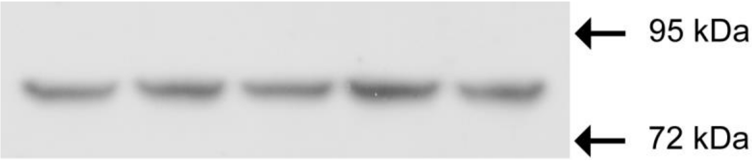

PI3K-3

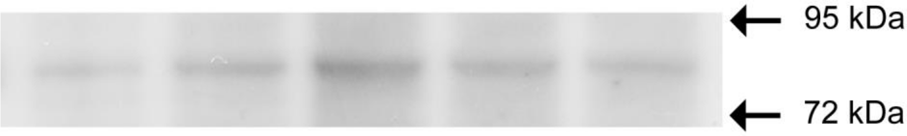

p-PI3K-1

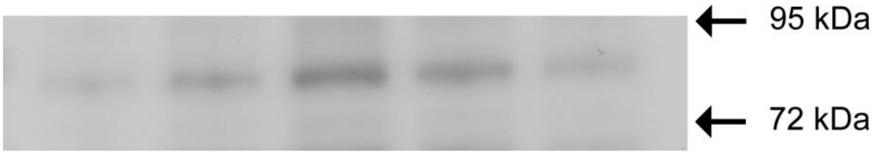

p-PI3K-2

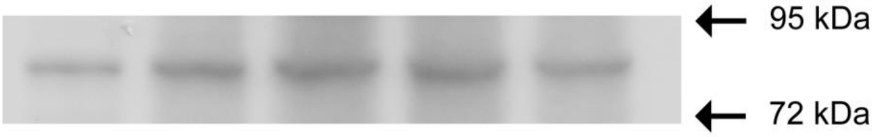

p-PI3K-3

昆药集团股份有限公司
KPC Pharmaceuticals, Inc.

检 验 报 告

检品名称：注射用血塞通（冻干）
检品批号：16BB205
规　　格：200mg*10支/盒*54盒/箱
产品数量：162件48盒（87960支）

生产日期：2016-02-02
有效期至：2019-01
报告日期：2016-02-18
检验依据：按照WS-10986(ZD-0986)-2002-2011Z和《中国药典》2015版标准

检验项目	标准规定	检验结果
【性状】	本品为类白色至淡黄色无定形粉末或疏松固体状物；味苦、微甘；有引湿性	本品为类白色疏松固体状物；味苦、微甘；有引湿性
【鉴别】液相色谱	应呈现于三七总皂苷对照提取物中R1、Rg1、Re、Rb1、Rd色谱峰保留时间相同的色谱峰	符合规定
【检查】不溶性微粒	每个供试品容器中：含10um及10um以上微粒不得过6000粒，含25um及25um以上微粒不得过600粒	每个供试品容器中：含10um及10um以上微粒373粒，含25um及25um以上微粒13粒
pH值	5.0~7.0	5.9
溶液的颜色	溶液应澄清；与4号黄色标准比色液比较，不得更深	溶液澄清；浅于2号黄色标准比色液
干燥失重	≤5.0%	0.38%
蛋白质	应符合规定	符合规定
鞣质	应符合规定	符合规定
树脂	应符合规定	符合规定
草酸盐	应符合规定	符合规定
钾离子	应符合规定	符合规定
炽灼残渣	≤0.5%	0.05%
异常毒性	应符合规定	符合规定
热原	应符合规定	符合规定
无菌	阳性管在48~72小时内应长菌，其余管培养14天不得长菌	符合规定
溶血与凝聚	应符合规定	符合规定
装量差异	±7%	±7%
可见异物	应符合规定	符合规定
指纹图谱	应符合规定	符合规定
重金属及有害元素残留	应符合规定	符合规定
【含量】（标示量计）三七皂苷R1	6.0%~16.0%	9.8%
【含量】（标示量计）人参皂苷Rg1	28.0%~50.0%	38.4%
【含量】（标示量计）人参皂苷Re	2.5%~7.0%	5.4%
【含量】（标示量计）人参皂苷Rb1	26.0%~40.0%	34.8%
【含量】（标示量计）人参皂苷Rd	3.5%~12.0%	4.7%
【含量】（标示量计）R1+Rg1+Rb1+Re+Rd	80.0%~115.0%	93.0%
结论：	本品按照WS-10986(ZD-0986)-2002-2011Z和《中国药典》2015版标准检测，结果：符合规定	

合格品

检验者：丁文纯,耿礼景,任颖达,申鸿,王政	复核者：罗兴芳,唐一凡,张珍妮,朱菊仙
审核意见：符合规定	检验监督QA：胡萍娜 2016.02.24
审核意见：符合要求	生产监督QA：[illegible] 2016.02.25
放行责任人：胡云 2016.02.25	

昆药集团股份有限公司检验专用章
